# Chromosome 19 miRNA cluster and CEBPB expression specifically mark and potentially drive triple negative breast cancers

**DOI:** 10.1371/journal.pone.0206008

**Published:** 2018-10-18

**Authors:** Goodwin G. Jinesh, Elsa R. Flores, Andrew S. Brohl

**Affiliations:** 1 Department of Molecular Oncology, H. Lee Moffitt Cancer Center & Research Institute, Tampa, FL, United States of America; 2 Sarcoma Department, H. Lee Moffitt Cancer Center & Research Institute, Tampa, FL, United States of America and; 3 Department of Cutaneous Oncology, H. Lee Moffitt Cancer Center & Research Institute, Tampa, FL, United States of America; 4 Cancer Biology and Evolution Program, H. Lee Moffitt Cancer Center & Research Institute, Tampa, FL, United States of America; 5 Chemical Biology and Molecular Medicine Program. H. Lee Moffitt Cancer Center & Research Institute, Tampa, FL, United States of America; University of South Alabama Mitchell Cancer Institute, UNITED STATES

## Abstract

Triple negative breast cancers (TNBCs) are known to express low PGR, ESR1, and ERBB2, and high KRT5, KRT14, and KRT17. However, the reasons behind the increased expressions of KRT5, KRT14, KRT17 and decreased expressions of PGR, ESR1, and ERBB2 in TNBCs are not fully understood. Here we show that, expression of chromosome 19 miRNA cluster (C19MC) specifically marks human TNBCs. Low REST and high CEBPB correlate with expression of C19MC, KRT5, KRT14, and KRT17 and enhancers of these genes/cluster are regulated by CEBPB and REST binding sites. The C19MC miRNAs in turn can potentially target REST to offer a positive feedback loop, and might target PGR, ESR1, ERBB2, GATA3, SCUBE2, TFF3 mRNAs to contribute towards TNBC phenotype. Thus our study demonstrates that C19MC miRNA expression marks TNBCs and that C19MC miRNAs and CEBPB might together determine the TNBC marker expression pattern.

## Introduction

Chromosome-19 micro-RNA cluster (C19MC) is one of the largest miRNA cluster found in the human genome and it accommodates 46 micro RNAs (miRNAs) at ‘q’ arm of chromosome 19, band chr19q13.42 within a span of ~100kb[1]. C19MC miRNAs have been implicated in various cancer types such as hepatocellular carcinoma[2, 3], embryonal tumors with multilayered rosettes (ETMRs)[4], infantile hemangioma[5], testicular germ cell tumors[6], parathyroid tumors[7], and thyroid adenomas[8]. The major role of C19MC in human breast cancer is not known yet except a report that suggests the contribution of C19MC miR-519a to apoptosis resistance [9].

Human breast cancer has an estimated incidence of 257,910 new invasive cases and 40,610 estimated deaths in 2017 based on American Cancer Society, Inc., Surveillance Research, 2017[10]. Breast cancer is classified into different molecular sub-types based on receptor expression which are luminal-A, luminal-B, HER’s-2-enriched (*ERBB2*+), triple negative breast cancers (TNBCs)[10] and normal-like breast cancers[11]. Luminal-A tumors express estrogen receptor (ER/*ESR1*) and progesterone receptor (PR/*PGR*) but not HER2 receptor and accounts for 71% of the breast cancers but are slow growing and less aggressive[10]. Luminal-B tumors express ER, PR and HER2 receptors and accounts for about 12% of the breast cancers but are more aggressive than luminal-A sub-type cancers[10]. HER-2-enriched tumors (ERBB2+) express high HER-2 levels but do not express ER or PR and accounts for approximately 5% of breast cancers with aggressive spreading behavior[10]. TNBCs do not express or under-express ER, PR or HER2 and represents 12% of the breast cancers and about 75–90% of basal-like breast cancers[10, 12] that are peculiarly therapy resistant in nature[13, 14]. TNBCs also have elevated expression of KRT5, KRT14 and KRT17 as markers[12]. TNBCs and *ERBB2*+ tumors are highly similar in terms of marker expression except ERBB2, and the functional status of *ERBB2*/HER2 receptor distinguishes *ERBB2*+ tumors from TNBCs[15]. Loss of *TFF3* expression marks the TNBCs from cells with functional *ERBB2*+[15] and hence *TFF3* can be an additional marker used to distinguish *ERBB2*+ tumors from TNBCs.

Gene expressions are powerfully regulated by enhancers (which usually bear the histone acetylation mark, H3K27Ac) and enhancers are known to regulate breast cancer cells in a molecular-sub-type dependent fashion[16]. Enhancers are recognized by CCAAT/Enhancer Binding Proteins (CEBPs) to boost enhancer-driven transcription. RE1-silencing transcription factor (REST) expression is lost in TNBCs[17] and its degradation is known to promote TNBCs[18]. However, the mechanism behind the role of REST degradation in promoting TNBCs and the link between enhancer activation, and C19MC expression are not understood to date.

Using human breast cancer patient data here we show that, C19MC miRNAs are the most tightly co-expressed miRNA set among all miRNAs that are expressed, and their high expression tightly correlates with basal-like TNBCs. A high CCAAT/Enhancer Binding Protein-β (CEBPB^high^) and low REST (REST^low^) expression profile positively correlates with C19MC expression and TNBCs. C19MC miRNA expression negatively correlates with PGR, ESR1 and ERBB2 mRNA expression and positively correlates with KRT5, KRT14 and KRT17 mRNA expression. C19MC miRNAs can target REST mRNA to potentially relieve the repressive effects of REST on C19MC or at CEBPB enhancer and can target PGR, ESR1, ERBB2, GATA3, SCUBE2, and TFF3 to contribute towards triple negative phenotype. Thus our study demonstrates that, a REST versus CEBPB plus C19MC miRNA expression marks TNBCs and that C19MC miRNAs plus CEBPB may potentially drive expression of key markers of TNBCs.

## Materials and methods

### The Cancer Genome Atlas (TCGA) and other online resources

TCGA miRNASeq, RNASeq, 450k methylation array data [level 3 TCGA data for methylation arrays (450k Infinium chip) and expression (Illumina HiSeq RNAseq, summarized by exons and genes, hg19 genome] were from (https://gdac.broadinstitute.org/) or accessed through TCGA wanderer (http://maplab.imppc.org/wanderer/). For non-CpG methylation maps within C19MC (and corresponding C19MC miRNA match), UCSC genome browser (https://genome.ucsc.edu/cgi-bin/hgGateway) and TCGA wanderer sites were used. For UCSC genome browser, hg19 genome assembly was used throughout the study. Multi-cell line ChIPSeq data was from ENCODE (https://www.encodeproject.org/). Mature miRNA sequences were from miRBase (http://www.mirbase.org/). RefSeq mRNAs for REST (Accession: NM_005612.4), PGR (Accession: NM_001202474.3), ESR1 (Accession: NM_000125.3), ERBB2 (Accession: NM_004448.3), GATA3 (Accession: NM_001002295.1), SCUBE2 (Accession: NM_020974.2), XBP1 (Accession: NM_005080.3), and TFF3 (Accession: NM_003226.3) were from GenBank (https://www.ncbi.nlm.nih.gov/genbank/).

### Correlation analyses

Correlation plots were generated using package ‘corrplot’ 0.84 (was built under R version 3.4.4) Correlation plots with C19MC labels were generated using integrated miRNASeq (C19MC miRNA sum), and 450k methylation array probe β values or RNASeq (rpm) (please see below for integration method). The data were log transformed to the base of 10 before generating matrix table in R. The scripts are available in supplementary materials.

### C19MC-based grouping of breast cancer patients

Gene expression by miRNASeq dataset of breast invasive carcinoma (including normal, and primary breast cancers) was processed to get cumulative C19MC miRNA expression of all 46 miRNA genes (*MIR498*, *MIR512-1*, *MIR512-2*, *MIR515-1*, *MIR515-2*, *MIR516A1*, *MIR516A2*, *MIR516B1*, *MIR516B2*, *MIR517A*, *MIR517-B*, *MIR517C*, *MIR518A1*, *MIR518A2*, *MIR518B*, *MIR518C*, *MIR518D*, *MIR518E*, *MIR518F*, *MIR519A1*, *MIR519A2*, *MIR519B*, *MIR519C*, *MIR519D*, *MIR519E*, *MIR520A*, *MIR520B*, *MIR520C*, *MIR520D*, *MIR520E*, *MIR520F*, *MIR520G*, *MIR520H*, *MIR521-1*, *MIR521-2*, *MIR522*, *MIR523*, *MIR524*, *MIR525*, *MIR526A1*, *MIR526A2*, *MIR526B*, *MIR527*, *MIR1283-1*, *MIR1283-2*, *and MIR1323*). Metastatic samples were excluded because the number of metastasis samples was too small. Again the patients who do not have miRNASeq data also were excluded. Normal breast samples were used to set a cut-off for overexpression, which we defined as >2 standard deviations above the mean. A cut-off value of 34.02 was set based on the normal dataset (mean = 12.2, standard deviation = 10.91 and n = 87). There were 67 breast cancer specimens which expressed cumulative C19MC miRNA value above the cut-off and designated as C19MC^high^ group. We then sorted the primary breast cancer patients based on cumulative C19MC expression values and selected equal number of patients (67) who had least or no cumulative C19MC expression to form the C19MC^low^ group.

### Integrated miRNASeq and RNASeq analysis of C19MC^high^ and C19MC^low^ breast cancer patients

RNASeq dataset of breast invasive carcinoma was matched to the miRNASeq dataset to examine the gene expression by cluster v3.0 using SD gene vector = 5000 to filter genes. Resulting 408 genes that passed the filter were log transformed, genes and arrays median centered, genes were clustered by correlation centered metric and subjected to average linkage. The clusters were visualized using Treeview v1.1.6r4. Genes that most strikingly showed differential expression between C19MC^low^ and C19MC^high^ sets were grouped and labelled as cluster-1 and cluster-2.

### Integrated miRNASeq and 450k methylation array analysis

#### CpG island

Human breast cancer 450k methylation array dataset targeting pre-C19MC region (C19MC upstream sequence) of chromosome-19 (hg19 coordinates: Chr19:54,100,000-54-169,000) and the CpG island located within (hg19 coordinates between: Chr19:54,151,000-54-152,000 with 4 probes for CpG island: probes cg15096240, cg09065632, cg16187069, cg00886824 designated as P1, P2, P3 and P4 respectively) were matched to miRNASeq dataset of breast invasive carcinoma to examine the correlation of hypomethylation of CpG island to C19MC expression. Methylation beta values of patients for each probes were sorted based on values and the curves that start falling drastically from the core of the sample set for majority of the probes (value: 0.9) was set as cut-off value that discriminate hypomethylation (<0.9) from hypermethylation (>0.9).

#### Full C19MC

Human breast cancer 450k methylation array dataset targeting C19MC region (full C19MC) of chromosome-19 (hg19 coordinates: Chr19:54,169,000-54-265,000) with 85 focal non-CpG methylation probes (includes one non-miRNA locus also) corresponding to 39 of C19MC miRNAs by loci were matched to miRNASeq dataset of breast invasive carcinoma to examine the correlation of hypomethylation of non-CpG island hypomethylation to individual matching C19MC expression.

### H3K27Ac mark, enhancer, and transcription factor binding site bioinformatics

UCSC genome browser was used to identify the transcriptional regulators such as H3K27Ac marks, chromatin structure regulators, repressors and other transcription factor binding sites within C19MC start site (Chr19:54,167,000-54-177,000), full C19MC (Chr19:54,169,000-54-265,000), *CEBPB* (chr20:48,806,593–48,811,000), *KRT5* (chr12:52,905,359–52,920,243), *KRT14* (chr17:39,735,000–39,746,000) and *KRT17* (chr17:39,772,000–39,785,000) genes. The genes were focused along with their upstream and internal regulatory elements using the hg19 coordinates above.

### REST/CEBPB/CTCF/TNBC-matched C19MC-based, and TNBC-based patient grouping

Human breast invasive carcinoma RNASeq patient dataset was sorted based on REST expression and REST^low^ and REST^high^ patients (n = 300 each) and was used to explore the expression of C19MC regulatory factors. A similar approach was used for CEBPB/CTCF-based patient grouping. Alternatively, a triple negative breast cancer patient group (n = 118) and a non-triple negative breast cancer patient group (n = 589) were sorted among the primary breast cancer patients who had datasets for miRNASeq, RNASeq and triple negative status. Patients who did not had datasets for all three (miRNASeq, RNASeq and triple negative status), were excluded from the analysis. When patients had equivocal receptor status by IHC, FISH-based receptor positivity was used to judge the receptor status. Patients who lack details on all three of the receptor status details (ER/HER2/PR) were excluded from both TNBC and non-TNBC groups. Patients who had at least one receptor positivity but lack details on other two receptors were included in non-TNBC groups. For TNBC-matched C19MC-based patient grouping the initial miRNASeq plus RNASeq integrated dataset (see section above: C19MC-based grouping of breast cancer patients) was matched to TNBC status and for each group 63 patients were chosen based on the availability of TNBC status data in one of the groups.

### C19MC miRNA target mapping

For C19MC miRNA target mapping mature miRNA sequences of miR-520g (Accession: MI0003166) from miRBase were converted to reverse complement sequence and matched to target RefSeq mRNAs (transcript variant-1 for target mRNAs were chosen if more than one RefSeq sequences are available: See section “The Cancer Genome Atlas (TCGA) and other online resources” for accession numbers). 6-mer to 10-mer matches were categorized based on the location of matches within mRNAs such as 5’-UTR, coding sequence, and 3’-UTR and the match locations were plotted using Circa software (OMGenomics). Among the matches, the presence of seed sequence match(es) was/were verified.

### REST ChIPSeq data analysis

K562(ENCFF465GUK), Panc-1(ENCFF520YQP), A549(ENCFF804EKT), PFSK-1(ENCFF471PQR), GM12878(ENCFF430KUN), HepG2(ENCFF322BAY), SK-N-SH (ENCFF709QNF), and HeLaS3(ENCFF553EXE) cell line ChIPSeq data (BED files) were used to analyze REST binding within C19MC and surrounding region. ENCODE cell line ChIPSeq data for REST ChIPSeq was trimmed to focus the following regions within chromosome-19 surroundings (hg19): 69 kb pre-C19MC region (Chr19:54,100,000-54-169,000), 96 kb full C19MC region (Chr19:54,169,000-54-265,000), and 35 kb post-C19MC region. Hg38 data were converted to hg19, before trimming based on hg19 coordinates. A combined REST binding map in the C19MC and surrounding region was then plotted using Circa software (OMGenomics).

### Statistical analyses

Frequency distribution box-plots and statistical analyses were done using Graphpad Prism software (v7.04; La Jolla, CA, USA). Box-plots are of 10–90 percentile type with aligned outliers with 75% transparency. For group versus group statistical significance analysis (box-plots), unpaired, non-parametric Mann-Whitney test was used. For paired correlation analysis R-studio software (Version 1.1.423) with Corrplot 0.84 package was used. Please see the section “Correlation plots, scripts, color code and statistical significance” for more details on R-statistics. Throughout the study the p-value of 0.05 was considered significant and for frequency distribution box-plots, p-values <0.001 were considered as robust significance.

## Results

### C19MC overexpression specifically marks triple negative breast cancers

**Fig 1:**
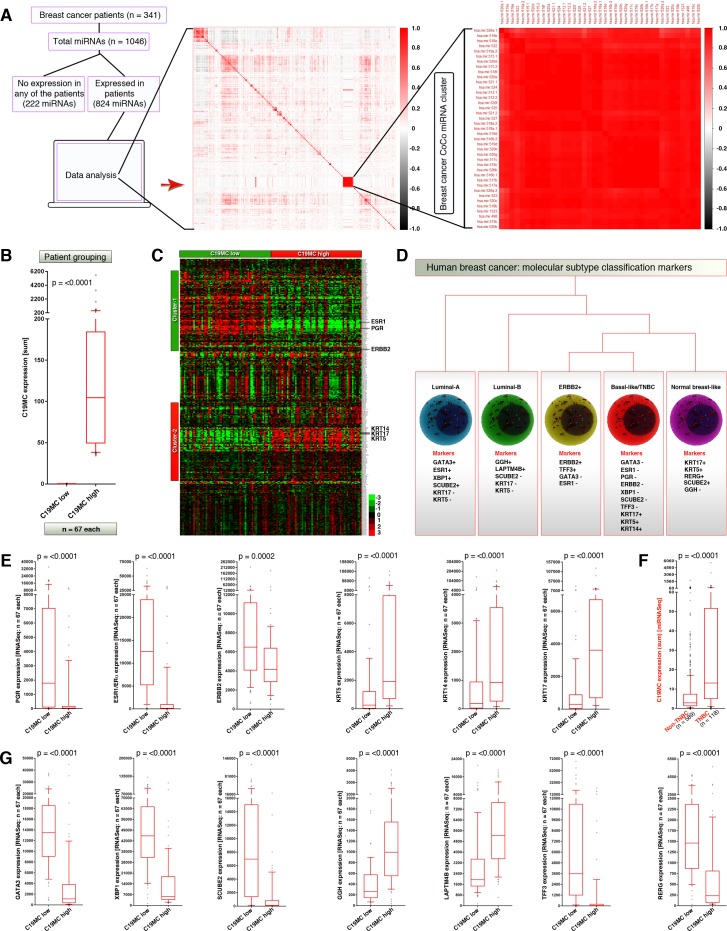
C19MC miRNAs, the most correlatively co-expressed miRNA set in breast cancer specifically marks triple negative breast cancers. **A,** Correlation analysis of all expressed miRNAs in human breast cancer to show that C19MC miRNAs are the most correlatively co-expressed (CoCo) miRNAs **B,** C19MC-based breast cancer grouping: C19MC^high^ and C19MC^low^ **C,** RNASeq heatmap analysis of miRNASeq matched C19MC^high^ and C19MC^low^ groups (n = 67 each) **D,** Schematic showing the classification of breast cancer into molecular sub-types (based on published reports[11, 15, 53]). **E,** Expression of triple negative markers at mRNA level, in C19MC-based groups **F,** Cumulative C19MC miRNA expression in TNBC-based groups **G,** Expression levels of non-TNBC markers at mRNA level in C19MC-based groups. All p-values for box-plots were obtained using Mann-Whitney test.

To understand the expression pattern of C19MC miRNAs in human breast cancer, we subjected the miRNASeq TCGA data for correlation analysis. Among 1046 miRNAs in 341 patients, 222 miRNAs were not expressed in any of the patients (Fig 1A). Correlation cluster analysis of remaining 824 miRNAs revealed that, 39/46 of the C19MC miRNAs (HSA-MIR-498, 512–1, 512–2, 515–1, 515–2, 516B-1, 516B-2, 517A, 517B, 517C, 518A-1, 518A-2, 518B, 518C, 518D, 518E, 518F, 519A-2, 519C, 519D, 519E, 520A, 520B, 520C, 520D, 520F, 520G, 520H, 521–1, 521–2, 522, 523, 524, 525, 526A-1, 526A-2, 526B, 527, and 1323) formed a tight and positively correlated coexpressed cluster (CoCo cluster) whereas the remaining 7/46 of the C19MC miRNAs (HSA-MIR-516A-1, 516A-2, 519A-1, 519B, 520E, 1283–1, and 1283–2) formed separate or marginally lesser positively correlated mini-clusters (Fig 1A). Of note, all C19MC miRNA correlations were statistically significant (p = <0.05) with strong correlation scores revealed by deep red color in the heatmap (Fig 1A). No other miRNAs formed clusters as dense and large as C19MC, demonstrating that, C19MC CoCo cluster is the most co-expressed miRNA set among all miRNAs in human breast cancer and that individual miRNAs within this cluster are likely under the same transcriptional regulation.

To gain insights into the degree of C19MC expression in human breast cancers we segregated the TCGA miRNASeq data of breast invasive carcinoma primary tumors into C19MC^high^ and C19MC^low^ groups using cumulative C19MC miRNA expression with a cut-off for elevated expresion based on the matched normal breast specimens. Cumulative C19MC miRNA expression was used rather than individual 46 miRNAs as the mature miRNAs from this cluster show high degree of sequence similarity (data not shown). As designed, the C19MC^high^ and C19MC^low^ groups were well segregated in terms of C19MC expression with a statistical significance of p = <0.0001 (Fig 1B).

To investigate the biological consequences of high C19MC expression, we integrated the TCGA RNASeq dataset with the miRNASeq-based C19MC^high^ and C19MC^low^ groups (n = 67 each) and examined the group-wise gene expression changes. Integrated miRNASeq and RNASeq analysis had revealed that C19MC-relevant sets of genes were associated with two main clusters. Cluster-1 had downregulated genes in C19MC^high^ group and Cluster-2 had upregulated genes in C19MC^high^ group (Fig 1C). Interestingly, the C19MC^high^ group harbored significantly downregulated expression of PGR, ESR1 and ERBB2 mRNAs compared to C19MC^low^ group, the candidate “negative markers” of triple negative breast cancers (Fig 1C–1E)[12, 19]. On the other hand, the C19MC^high^ group harbored significantly upregulated expression of KRT5, KRT14 and KRT17 mRNAs compared to C19MC^low^ group, the candidate “positive markers” of basal-like triple negative breast cancers (TNBCs) (Fig 1C–1E)[12]. We validated this result using a reciprocal approach by sorting primary breast cancer patients into TNBCs and non-TNBCs and examined the cumulative expression of C19MC. TNBCs expressed significantly higher levels of C19MC miRNAs compared to non-TNBCs (p<0.0001) confirming that, high expression of C19MC marks TNBCs (Fig 1F).

To understand whether elevated C19MC expression is specific to TNBCs or also occurs in other molecular sub-types of breast cancers, we examined the expression of additional sub-type markers in C19MC^high^ and C19MC^low^ groups. Strikingly, C19MC^high^ tumors expressed significantly lower GATA3, XBP1, SCUBE2, TFF3, and RERG compared to C19MC^low^ group, to show that C19MC^high^ tumors are not indeed luminal-A, ERBB2+, and normal breast-like sub-types (Fig 1D and 1G). Although C19MC^high^ tumors expressed significantly higher GGH and LAPTM4B compared to C19MC^low^ group (Fig 1G), high expression of KRT5 and KRT17 discriminated C19MC^high^ tumors from luminal-B sub-type (Fig 1D and 1G). Therefore high C19MC expression is specific to TNBCs. Taken together these results demonstrate that, C19MC is the most co-expressed miRNA set among all miRNAs in human breast cancer and their elevated expression specifically marks triple negative breast cancers.

### C19MC expression does not correlate with hypomethylation of upstream CpG-island and weakly correlates to hypomethylation of non-CpG sites within C19MC

**Fig 2:**
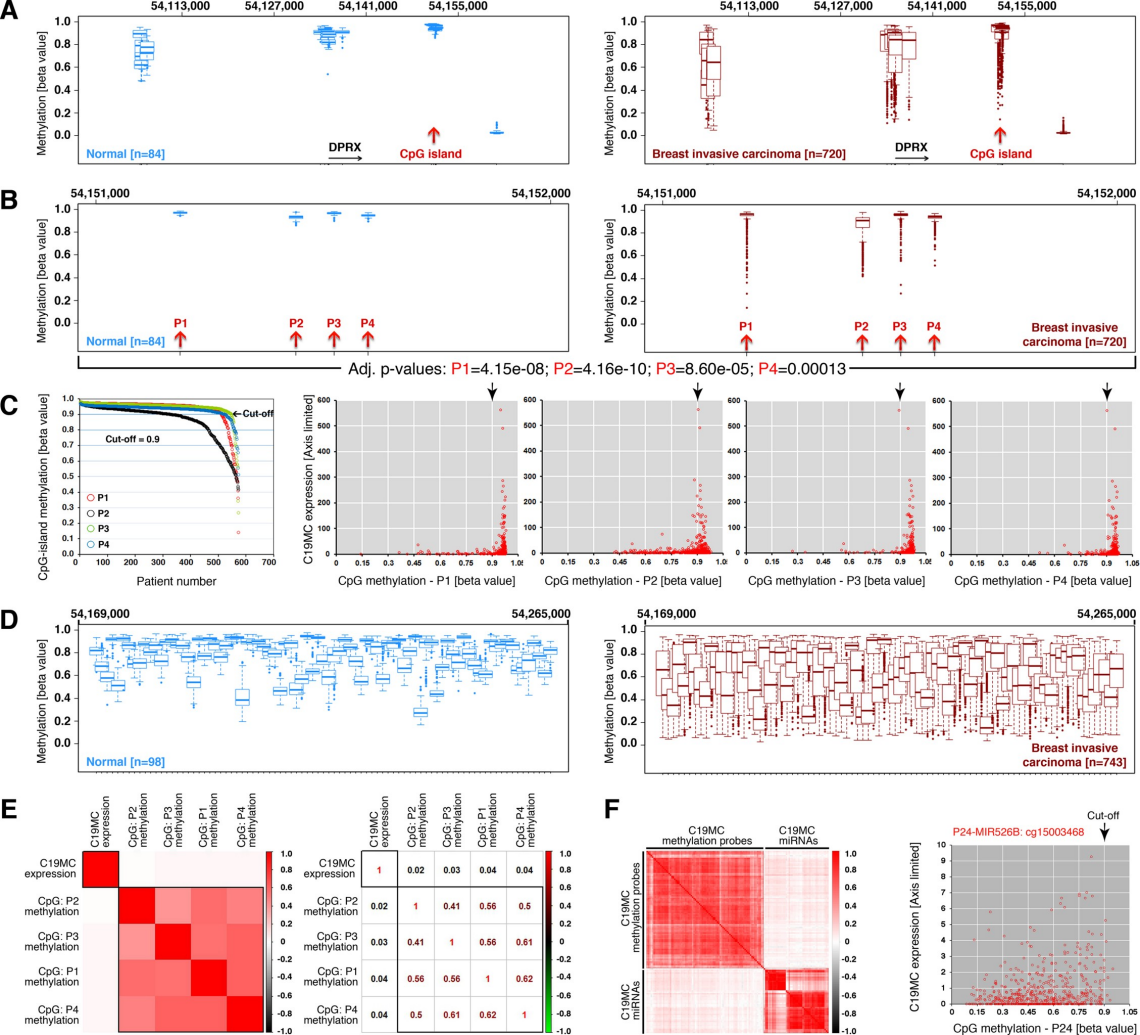
C19MC expression does not correlate with hypomethylation of upstream CpG-island but feebly correlates to hypomethylation of non-CpG sites within C19MC. **A,** Comparative methylation of pre-C19MC region between normal and breast invasive carcinoma using TCGA wanderer 450k methylation data. The gene DPRX was used to locate the region. Red arrows indicate the CpG island location. **B,** Comparative methylation of CpG-island region between normal and breast invasive carcinoma using TCGA wanderer 450k methylation data. The four probes were named P1-4. The adjusted p-values for all four probes were indicated below. **C,** Determining the cut-off values to stratify hypo versus hypermethylation of CpG-island probes (left) and correlation of C19MC expression to methylation status of CpG-island probes using XY-scatter plot. Arrows indicate the cut-off value. **D,** Comparative non-CpG methylation of C19MC region between normal and breast invasive carcinoma using TCGA wanderer 450k methylation data. **E,** Correlation heatmap showing lack of correlation between C19MC expression and CpG-island methylation status (left) with correlation co-efficiency values (right). **F,** Correlation heatmap showing weak correlation between C19MC expression and non-CpG methylation status (left): a single plot with most correlation to hypomethylation was shown (right) whereas most plots were showing weaker correlation.

To understand the mechanism how C19MC expression is upregulated in a subset of breast cancers, we examined the methylation pattern of 69kb C19MC upstream region (Pre-C19MC: 54,100,000–54,169,000: hg19) using 450k methylation array data. Compared to normal samples, breast cancers have considerable hypomethylation in this region (Fig 2A), particularly, a subset of samples at the CpG-island within pre-C19MC region (Fig 2B). A closer examination revealed that all four probes (P1-4) within CpG-island exhibited hypomethylation in breast cancers but P2 had more hypomethylation compared to normal samples (Fig 2B). We then evaluated within breast cancer specimens whether hypomethylation correlates with C19MC miRNA expression (Fig 2C). We integrated the miRNASeq C19MC cumulative expression dataset with 450k methylation dataset and found that, CpG-island hypomethylation did not correlate to elevated C19MC expression suggesting that the C19MC upstream CpG-island is not the primary determinant for regulation of C19MC expression in human breast cancer (Fig 2C).

We next examined the C19MC region (54,169,000–54,265,000: hg19), as C19MC region harbors numerous non-CpG methylation sites (S1 Fig). Compared to matched normal samples, tumor samples had drastic hypomethylation within C19MC (Fig 2D) Correlation analysis of CpG island and C19MC non CpG methylation using integrated 450k methylation data set with miRNASeq C19MC dataset showed that CpG island hypomethylation did not correlate with C19MC expression (Fig 2E) whereas non-CpG hypomethylation weakly correlated with C19MC expression (Fig 2F). Together these data demonstrates that there is significant hypomethylation of both the pre-C19MC CpG-island and C19MC non-CpG-island methylation sites, however within breast cancers hypomethylation of either site does not correlate well to C19MC expression. These results suggest that additional factors are required for C19MC expression other than C19MC hypomethylation.

### C19MC start site harbors a strong enhancer with CEBPB site and CEBPB expression is elevated in TNBCs

**Fig 3:**
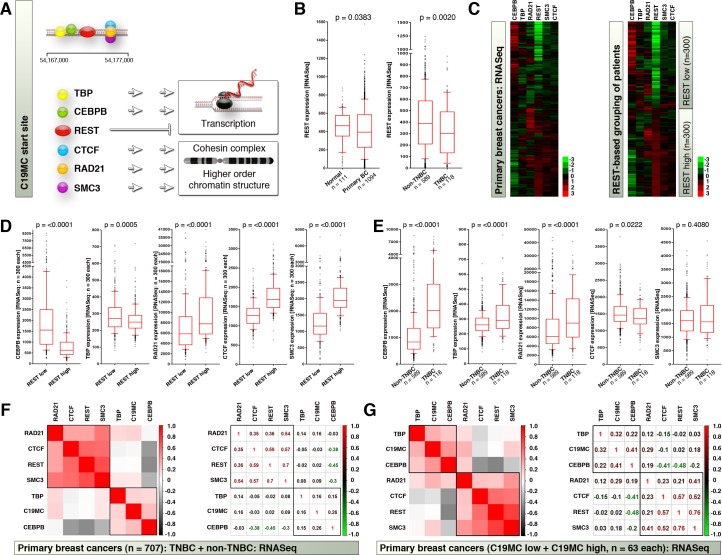
C19MC start site harbors a strong enhancer with CEBPB site and CEBPB expression is elevated in TNBCs. **A,** Schematic showing the C19MC start site binding factors and their functions. Also see S2A Fig. for UCSC genome browser data. **B,** Comparative RNASeq expression data for REST expression in normal versus primary breast cancers (left) and TNBCs versus non-TNBCs (right). **C,** RNASeq heatmaps showing the expression of C19MC start site binding factors in primary breast cancers (left) and REST^low^ and REST^high^ tumors (right). **D,** RNASeq distribution of C19MC start site binding factors in REST-based tumor groups. **E,** RNASeq distribution of C19MC start site binding factors in TNBC versus non-TNBC tumor groups. **F,** Correlation heatmap (left) and correlation co-efficiency (right) plots of C19MC start site binding factors (RNASeq) to C19MC expression (miRNASeq) in primary TNBC-based miRNASeq matched dataset. **G,** Correlation heatmap (left) and correlation co-efficiency (right) plots of C19MC start site binding factors (RNASeq) to C19MC expression (miRNASeq) in primary C19MC-based RNASeq matched dataset.

To understand additional factors that may regulate C19MC expression we examined the C19MC start site for transcription factor binding sites and enhancer elements using UCSC genome browser. Interestingly, the C19MC start site harbors a series of strong and weak H3K27Ac marks (Fig 3A and S2A Fig) indicating the existence of a possibility of enhancer-mediated regulation of C19MC. In support of this view, sequence-based CEBPB binding site very close to the strong H3K27Ac mark also located within C19MC start site (Fig 3A and S2A Fig). There also exists a REST binding site at the C19MC start region (Fig 3A and S2A Fig), where REST is a known repressor of transcription at non CpG-methylated regions[20]. C19MC start site also harbors binding sites for a group of factors TBP(TATA-Box Binding Protein/TFIID), CTCF, RAD21and SMC3 (Fig 3A) of these CTCF, RAD21 and SMC3 are higher order chromatin structure regulators by serving as cohesin core factors to insulate chromatin[21]. REST is the only transcriptional repressor[22] among this group and TBP and CEBPB are positive regulators of transcription[23, 24]. As REST is the only repressor among this group, and because REST degradation is known to promote TNBCs[18], we examined the status of REST, by comparing the normal and primary breast cancers and found that, REST is significantly downregulated in primary breast cancers compared to normal specimens (Fig 3B). In addition, REST expression is significantly lower in TNBCs compared to non-TNBCs (Fig 3B). We next sorted the RNASeq data of primary breast cancer patients based on REST^low^ and REST^high^ groups with 300 patients for each group and examined the expression patterns of TBP, CEBPB, REST, CTCF, RAD21 and SMC3 in addition to unsorted patient dataset (Fig 3C). The heatmaps indicated that REST expression is inversely correlated to CEBPB expression in primary breast cancers (Fig 3C). This is possibly because REST binding sites are located within CEBPB gene regulatory regions (S2B Fig). TBP and CEBPB were significantly increased in REST^low^ group whereas CTCF, RAD21and SMC3 were significantly reduced in REST^low^ group (Fig 3D).

In a TNBC-based patient RNASeq data classification, TBP and CEBPB were significantly increased in TNBCs along with RAD21, whereas CTCF and SMC3 did not show statistically significant difference (Fig 3E). Since RAD21 shown contrasting differences between TNBC-based, and REST-based classifications, we examined the correlation of the expression of C19MC start site factors by correlation heatmap clustering using TNBC-based, and C19MC miRNA expression-based (High and low) integrated datasets (RNASeq for C19MC start site factors, and miRNASeq for C19MC miRNAs). We found that, RAD21, CTCF, REST and SMC3 formed a tightly correlated cluster whereas TBP, C19MC miRNAs, and CEBPB formed a separate cluster where CEBPB exhibited a strong negative correlation to REST and CTCF expression (Fig 3F and 3G). Taken together these data indicate that C19MC start site harbors an enhancer and CEBPB is correlated to C19MC expression (where TBP is part of general transcription machinery) and suggested that REST^low^ levels could promote CEBPB expression to promote C19MC miRNA expression.

### REST can occupy across C19MC in a cell type-dependent manner and it is expression negatively correlates with CEBPB

**Fig 4:**
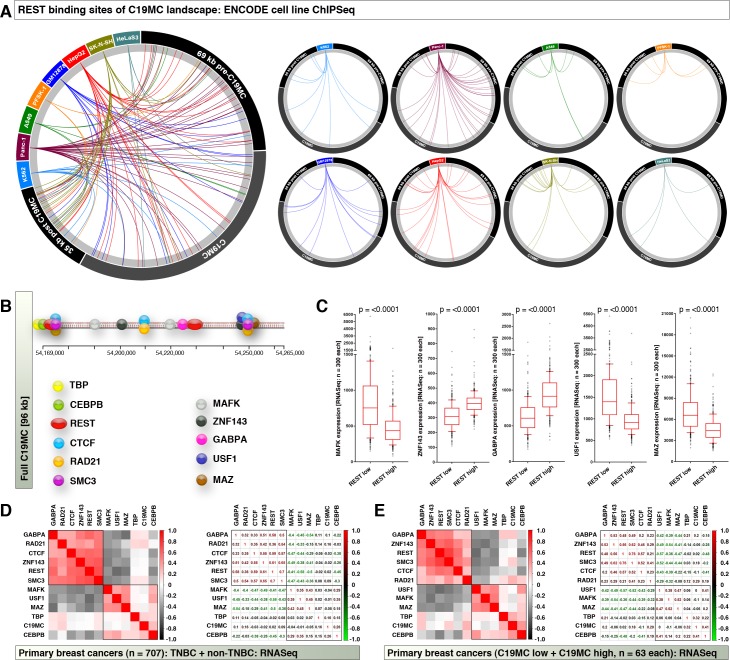
REST can occupy across C19MC in a cell type-dependent manner and it is expression negatively correlates with CEBPB. **A,** ENCODE ChIPSeq data (hg19) of 8 human cancer cell lines to show that REST can bind all across C19MC and surrounding genomic areas (left:combined map; right: individual cell line map)**. B,** Schematic showing transcription regulatory factor binding map of full C19MC region. Also see S3 Fig for UCSC genome browser data. **C,** RNASeq expression data of C19MC binding factors in REST-based tumor groups. **D,** Correlation heatmap (left) and correlation co-efficiency (right) plots of full C19MC binding factors (RNASeq) to C19MC expression (miRNASeq) in primary TNBC-based miRNASeq matched dataset. **E,** Correlation heatmap (left) and correlation co-efficiency (right) plots of full C19MC binding factors (RNASeq) to C19MC expression (miRNASeq) in primary C19MC-based RNASeq matched dataset.

REST degradation can promote TNBC status[18] therefore we examined the REST binding pattern in multi-cell line ChIP-Seq ENCODE data and found that, REST can bind (directly or indirectly) to pre-C19MC, C19MC and post-C19MC regions (54,100,000–54,169,000; 54,169,000–54,265,000; 54,265,000–54,300,000 respectively: hg19) in a cell type dependent manner (Fig 4A). In addition to C19MC start site binding factors described above, MAFK, ZNF143, GABPA, USF1 and MAZ can also bind within C19MC region (Fig 4B and S3 Fig). A REST-based grouping of RNASeq data revealed that, MAFK, USF1, and MAZ had significant increase in REST^low^ group suggesting these factors could play a role in C19MC transcription (Fig 4C). However, an examination by correlation heatmap clustering using integrated datasets (RNASeq for C19MC binding factors, and miRNASeq for C19MC miRNAs: groups sorted based on TNBCs vs. non-TNBCs or C19MC^high^ vs. C19MC^low^) revealed that, MAFK, USF1 and MAZ did not correlate to C19MC expression whereas GABPA and ZNF143 were clustered with REST and had higher correlation to REST than to C19MC expression (Fig 4D and 4E). Again, CEBPB is the factor which had strongest positive correlation to C19MC miRNA expression and negative correlation to the factors in REST containing negative regulatory cluster (Fig 4D and 4E). Taken together, these data shows that, CEBPB could be the central factor that might drive C19MC miRNA expression when REST is down.

### Activation of KRT5, KRT14 and KRT17 enhancers mark the functional CEBPB activation

**Fig 5:**
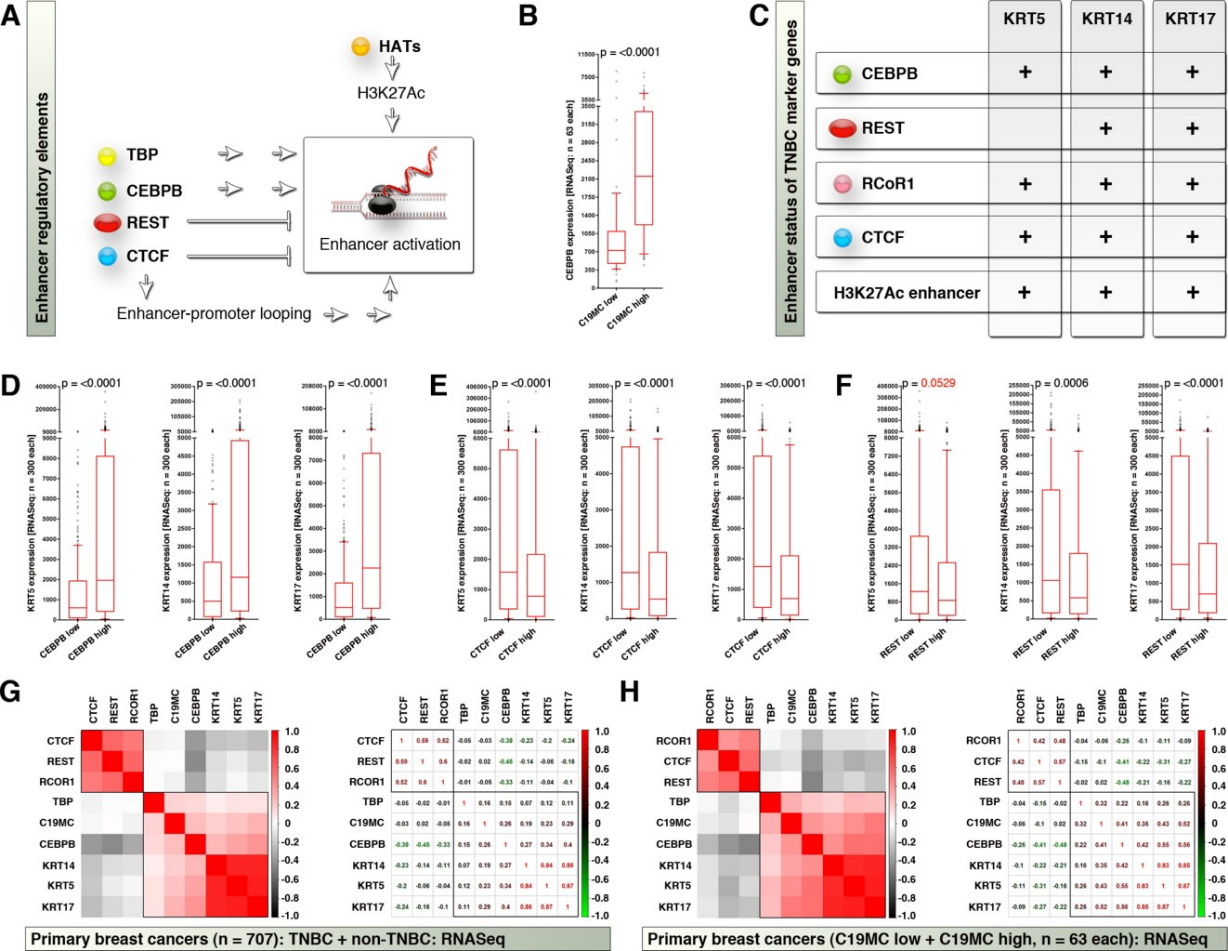
Activation of KRT5, KRT14 and KRT17 enhancers mark the functional CEBPB activation. **A,** Schematic showing the enhancer regulatory factors that map to C19MC start site. **B,** RNASeq data showing CEBPB expression in C19MC-based patient groups. Note: REST, RCoR1, and CTCF did not show statistical significance in this grouping and the data are not shown. **C,** Presence of enhancers and enhancer-regulatory factor binding sites, within KRT5, KRT14 and KRT17 gene-regulatory regions. **D-F,** CEBPB-based (D), CTCF-based (E) and REST-based (F) classification of RNASeq data, to show the expression differences of TNBC marker genes. **G,** Correlation heatmap (left) and correlation co-efficiency (right) plots of enhancer-regulatory factors (RNASeq) with C19MC-regulatory factors (miRNASeq) to TNBC marker expression (RNASeq) in primary “TNBC-based” miRNASeq matched dataset. **H,** Correlation heatmap (left) and correlation co-efficiency (right) plots of enhancer-regulatory factors (RNASeq) with C19MC-regulatory factors (miRNASeq) to TNBC marker expression (RNASeq) in primary “C19MC-based” RNASeq matched dataset.

While CEBPB and TBP can positively regulate transcription from enhancer elements[25, 26], REST and CTCF can negatively regulate transcription from enhancer elements[27, 28] where REST occupancy negatively correlate with H3K27Ac mark[29], and CTCF can have facultative role in facilitating enhancer loop formation[30] (Fig 5A). Among REST, CTCF, and CEBPB, CEBPB is the only member that was significantly enriched in C19MC^high^ group (Fig 5B; REST and CTCF data were not shown) and hence we investigated whether CEBPB is functional in triple negative breast cancers. First, CEBPB is significantly upregulated in TNBCs compared to other breast cancer molecular subtypes (p<0.0001) (Fig 3E). Although CEBPB can regulate the enhancers of a wide range of genes[31], we focused on the genes that are upregulated in C19MC^high^ tumors. Interestingly, all three TNBC marker genes upregulated in C19MC^high^ group viz., *KRT5*, *KRT14* and *KRT17* are regulated by enhancers and harbor H3K27Ac marks, CEBPB, and CTCF sites within the regulatory regions (Fig 5C and S4 Fig). *KRT14* and *KRT17* genes also had REST binding sites while *KRT5* had the REST co-repressor (RCoR1) binding sites (Fig 5C and S4 Fig). Therefore, a REST/CTCF versus CEBPB antagonistic regulation is viable in *KRT5*, *KRT14* and *KRT17* gene enhancers with lesser possibility for KRT5 gene as it lacks REST site. Classification of primary breast cancer RNASeq data based on high/low CEBPB/REST/CTCF revealed that, KRT5, KRT14 and KRT17 mRNAs were significantly upregulated in CEBPB^high^ and CTCF^low^ tumors (Fig 5D and 5E). In the case of REST^low^ tumors, only KRT14 and KRT17 mRNAs were significantly upregulated whereas, the KRT5 gene which does not have REST binding site and did not show a statistically significant difference (Fig 5F). Integrated miRNASeq and RNASeq data demonstrated that, CEBPB expression correlated with KRT5, KRT14 and KRT17 expression and negatively correlated to REST, CTCF and RCOR1 (Fig 5G and 5H). Therefore, the elevated expressions of KRT5, KRT14 and KRT17 mRNAs reflect the functional activation of CEBPB and repression or degradation of REST.

### CEBPB and C19MC miRNA expression marks PGR, ESR1, ERBB2, GATA3, XBP1, SCUBE2 mRNA downregulation

**Fig 6:**
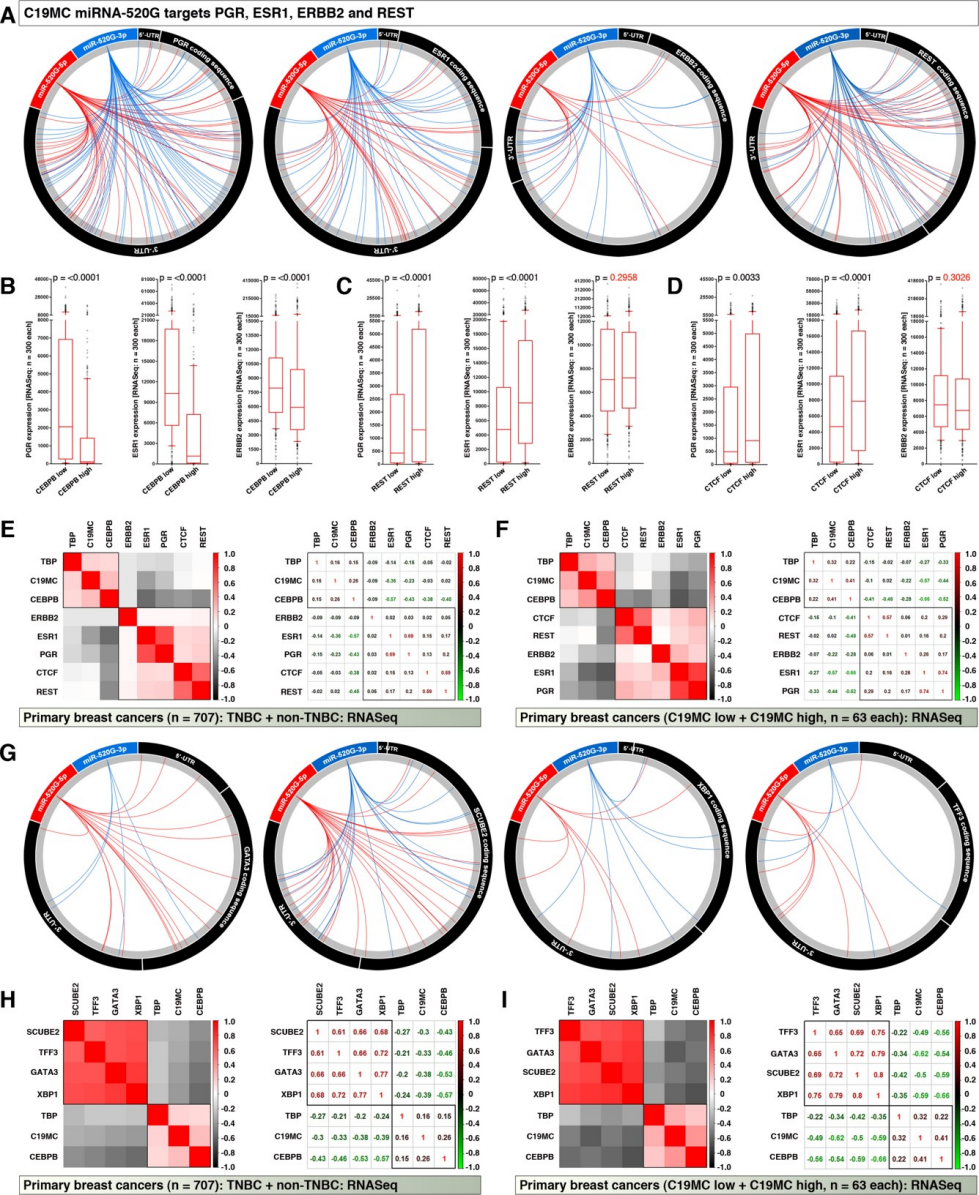
C19MC miRNA expression marks PGR, ESR1, ERBB2, GATA3, XBP1, SCUBE2 mRNA downregulation. **A,** C19MC miRNAs are capable of targeting PGR, ESR1, ERBB2 and REST mRNAs, as a case reverse complement sequences of mature miR-520g-5p and miR-520g-3p matches to target mRNAs (that includes seed sequence matches, 6-mers to 9mer/10-mers) were mapped to target mRNAs and plotted as circus plots. Note: target mRNAs are plotted in scale, but not miRNAs. **B-D,** RNASeq expression of triple-negative markers in CEBPB/REST/CTCF-based tumor groups (Note: these marker expressions in C19MC-based groups were shown in Fig 1E). **E,** Correlation heatmap (left) and correlation co-efficiency (right) plots of triple negative markers, and C19MC regulatory factors (RNASeq) with C19MC (miRNASeq) in primary “TNBC-based” miRNASeq matched dataset. Note the negative correlation (grey). **F,** Correlation heatmap (left) and correlation co-efficiency (right) plots of triple negative markers, and C19MC regulatory factors (RNASeq) with C19MC (miRNASeq) in primary “C19MC-based” RNASeq matched dataset. Note the negative correlation (grey). **G,** C19MC miRNAs are capable of targeting GATA3, SCUBE2, XBP1, and TFF3 mRNAs: as a case, reverse complement sequences of mature miR-520g-5p and miR-520g-3p matches to target mRNAs (that includes seed sequence matches, 6-mers to 9mer/10-mers) were mapped to target mRNAs and plotted as circus plots. Note: target mRNAs are plotted in scale, but not miRNAs. (Note: these marker expressions in C19MC-based groups were shown in Fig 1G) **H,** Correlation heatmap (left) and correlation co-efficiency (right) plots of non-TNBC breast cancer sub-type markers, and core C19MC regulatory factors (RNASeq) with C19MC (miRNASeq) in primary “TNBC-based” miRNASeq matched dataset. Note the negative correlation (grey). **I,** Correlation heatmap (left) and correlation co-efficiency (right) plots of non-TNBC breast cancer sub-type markers, and core C19MC regulatory factors (RNASeq) with C19MC (miRNASeq) in primary “C19MC-based” RNASeq matched dataset. Note the negative correlation (grey).

While the REST versus CEBPB expression pattern explains the expression of C19MC miRNA expression, we were interested to investigate why the triple negative marker mRNAs PGR, ESR1 and ERBB2 are under-expressed in C19MC^high^ group. We examined whether C19MC miRNAs can target PGR, ESR1 and ERBB2 RNAs. For this investigation we used miR-520G-5p and miR-520G-3p mature miRNA sequences and found that, these mature miRNAs can potentially target PGR, ESR1 and ERBB2 mRNAs as mature miRNAs including seed sequences match to reverse complement sequences of these mRNAs (Fig 6A). Furthermore, miR-520G-5p and miR-520G-3p mature miRNAs can potentially target REST mRNA for degradation (Fig 6A). As expected, CEBPB^high^ tumors expressed significantly lower PGR, ESR1 and ERBB2 mRNAs compared to CEBPB^low^ tumors (Fig 6B). This result is in agreement to the fact that, C19MC^high^ tumors express low levels of PGR, ESR1 and ERBB2 mRNAs (Fig 1E). Conversely, REST^low^ and CTCF^low^ tumors expressed significantly lower PGR and ESR1 mRNAs whereas ERBB2 did not reach a statistical significance (Fig 6C and 6D). Correlation heatmaps of integrated miRNASeq and RNASeq data revealed that, C19MC miRNA expression exhibited a strong negative correlation to PGR and ESR1 mRNA expression and to a lesser extent to ERBB2 mRNA and the degree of negative correlation was higher in C19MC-based dataset (Fig 6E and 6F). Furthermore, C19MC miRNAs can target GATA3, XBP1, SCUBE2 and TFF3 mRNAs (Fig 6G) suggesting a mechanism for downregulation of these non-TNBC markers in the C19MC^high^ group (Fig 1G). Correlation heatmaps of integrated miRNASeq and RNASeq data revealed that, C19MC miRNA expression exhibited a strong negative correlation to GATA3, XBP1, SCUBE2 and TFF3 mRNA expression and the degree of negative correlation was higher in C19MC-based dataset (Fig 6H and 6I). Taken together these data demonstrate that CEBPB and C19MC miRNA expression marks PGR, ESR1, ERBB2, GATA3, XBP1, SCUBE2, and TFF3 downregulation and therefore contribute to the TNBC phenotype.

## Discussion

TNBCs are the most aggressive form of breast cancer and shows significant therapy resistance. While cancer stem cells can gain therapy resistance primarily through apoptosis resistance[32–44] and resistance to immune surveillance[36, 45, 46], TNBCs utilize both immune evasion[47] and apoptosis resistance by displaying cancer stem cell properties[14]. In breast cancer one of the C19MC miRNA miR-519a was shown to offer apoptosis resistance in response to tamoxifen[9]. Apoptosis evasion and drug resistance are primarily mediated through evading p53-dependent transcriptional program[36]. In colon cancer cells, p53-dependent suppression of C19MC miRNA miR-520g expression is required to overcome apoptosis resistance [48]. TNBCs are being shown to influence the survival outcome by multiple studies[49–52]. Therefore, understanding the core biology behind TNBCs is of paramount importance to effective utilization of therapeutic measures or to develop new targeted therapies.

**Fig 7:**
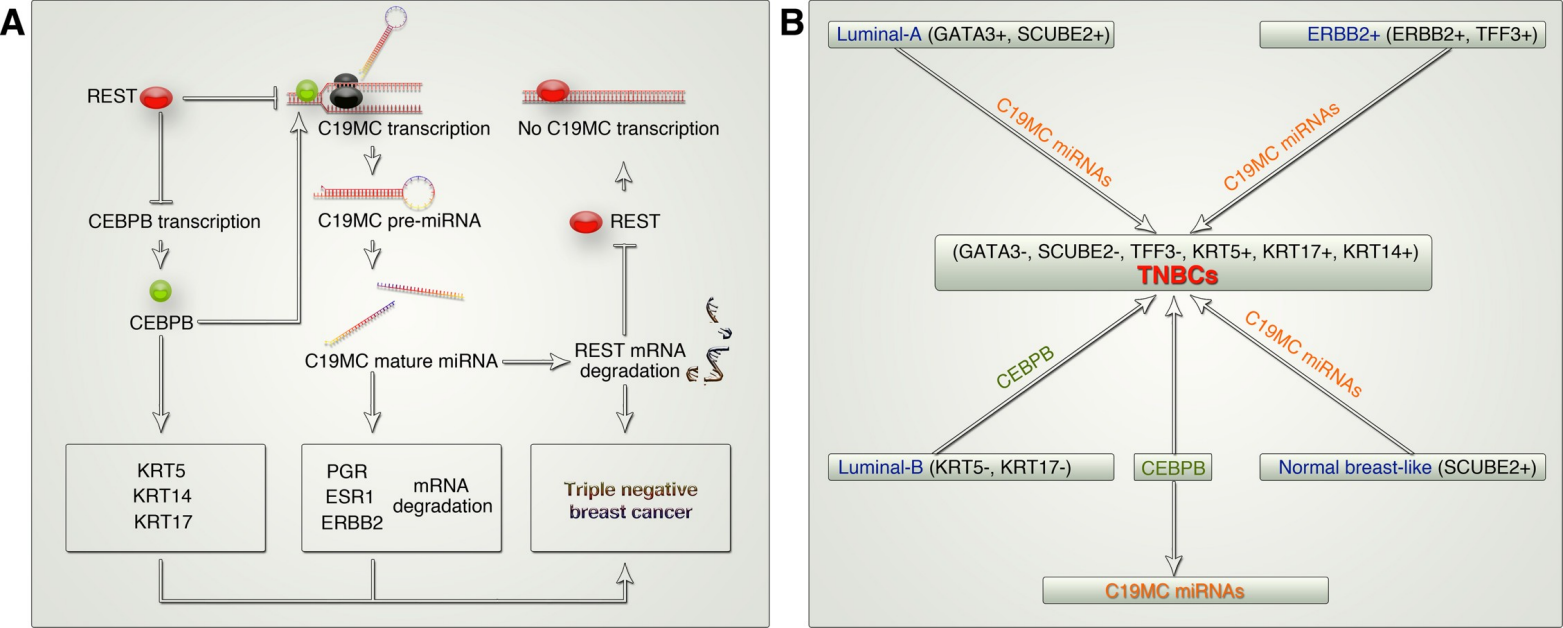
CEBPB and C19MC mark TNBCs and potentially drive TNBCs from other molecular sub-types of breast cancer. **A,** Schematic showing how CEBPB and REST play antagonistic roles in C19MC transcription to further regulate TNBC markers and REST mRNA to promote the triple negative status. **B,** Schematic showing how CEBPB and C19MC miRNAs act in concert to potentially drive TNBCs from other molecular sub-types of breast cancer.

It is well characterized that, KRT5, KRT14, and KRT17 are positive markers, whereas PGR, ESR1, and ERBB2 are negative markers for TNBCs[12]. However, it is not known why such marker expression specifically occurs in TNBCs. Here we for the first time show strong evidence based on integrated clinical and genetic information that, C19MC expression specifically marks TNBCs and that, REST^low^ status might drive the expression of KRT5, KRT14, and KRT17 through CEBPB-dependent enhancer activation, and target PGR, ESR1, and ERBB2 mRNAs through C19MC miRNAs to potentially drive triple negative breast cancers (Fig 7A). In this context REST degradation has been shown to promote triple negative status[18] but the possible mechanism downstream to REST degradation remained elusive to date. On the other hand, CEBPB has metabolic and immune evasive roles in TNBCs[52]. REST^low^ status can potentially relieve the repressive effects of REST on CEBPB to further regulate C19MC expression through CEBPB. At protein level, CEBPB has LAP and LIP isoforms (where LIP isoform serves as a negative regulator of LAP isoform), however the CEBPB-LAP isoform is active in TNBCs[52]. The high expression of KRT5, KRT14, and KRT17 reflects the expression and functional activation of CEBPB-LAP isoform and explains why C19MC expression is elevated in TNBCs.

The reverse complement sequence of C19MC miRNAs match GATA3, XBP1, SCUBE2 and TFF3 mRNAs suggesting the possibility that C19MC miRNA expression could degrade these mRNAs to potentially promote a TNBC phenotype rather than luminal-A, HER2+ and normal breast-like tumors. Additionally, KRT5 and KRT17 expression by CEBPB could create differentiation of TNBCs from luminal-B tumors, as luminal-B tumors differ from TNBCs by expressing KRT5 and KRT17 (Fig 7B). Thus our study for the first time proposes a regulatory axis of transcriptional factor and microRNA expression that drives a TNBC phenotype and differentiation from other molecular subtypes of breast cancer (Fig 7).

The strength of our study is that, we utilized a comprehensive and integrated approach of multiple datatypes including miRNASeq, ENCODE, RNAseq and 450k methylation data. Analysis of orthogonal sequencing and array technologies yielded consistent candidates such as C19MC, CEBPB, and REST as potential drivers/regulators of TNBCs. For example, high RAD21 correlates with TNBC (Fig 3E) but is clustered with negative regulators of C19MC (Fig 3F and 3G) and hence is not considered as a key player of C19MC positive regulation, whereas CEBPB consistently correlated with C19MC expression, TNBCs, and clustered with TBP (Fig 3E–3G) and hence is considered a potential driver of C19MC expression and TNBCs.

In summary, our study demonstrates that, a REST versus CEBPB plus C19MC miRNA expression marks TNBCs, and that C19MC miRNAs plus CEBPB in concert may potentially drive the TNBC phenotype. Further study is warranted to functionally validate these findings.

## Supporting information

S1 Fig CpG and non-CpG methylation patterns in the C19MC and flanking regions of chromosome-19. UCSC genome browser (hg19) was used to examine the methylation patterns in C19MC region. Green boxes indicate CpG islands and red border indicates the C19MC region packed with non-CpG methylation.
(TIF)

S2 Fig C19MC and CEBPB are regulated by CEBPB-dependent enhancers. (A) UCSC genome browser (hg19) was used to examine the C19MC region. Green arrows show the CEBPB and REST binding sites and red arrows show the H3K27Ac mark and strong enhancer. (B) UCSC genome browser (hg19) was used to examine the CEBPB gene. Red arrows show the REST binding sites. Note the strong enhancer mark H3K27Ac (blue to purple/pink peaks).
(TIF)

S3 Fig Transcription regulatory factor binding sites within at C19MC region. UCSC genome browser (hg19) was used to examine the transcription regulatory factor binding patterns in C19MC region (red box). Note the CEBPB and REST occupy the C19MC start site.
(TIF)

S4 Fig Transcription regulatory factor binding sites within KRT17, KRT14 and KRT5 genes. UCSC genome browser (hg19) was used to examine the transcription regulatory factor binding sites within within KRT17, KRT14 and KRT5 genes. Note the pink peaks (H3K27Ac marks) that indicating strong enhancer-mediated regulation of these genes. Red arrows indicate the REST binding sites or REST co-repressor RCoR1 binding sites.
(TIF)

S1 File Scripts for corrplots, color code and correlation efficiency. (DOCX)
