# Supplementary figures and images for: Chromosome 19 miRNA cluster and CEBPB expression specifically mark and potentially drive triple negative breast cancers
Source: PLoS One. 2018 Oct 18;13(10):e0206008. doi: 10.1371/journal.pone.0206008 (PMC6193703; doi:10.1371/journal.pone.0206008)

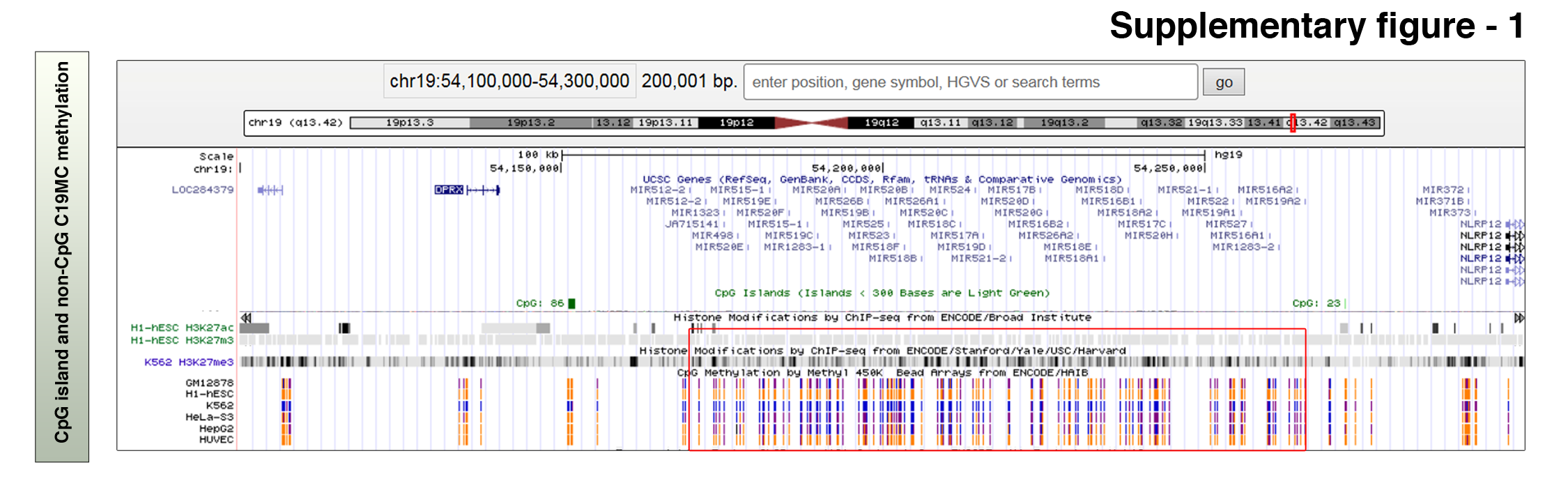

Supplement: S1 Fig — UCSC genome browser (hg19) was used to examine the methylation patterns in C19MC region. Green boxes indicate CpG islands and red border indicates the C19MC region packed with non-CpG methylation. (TIF) [file pone.0206008.s001.tif]

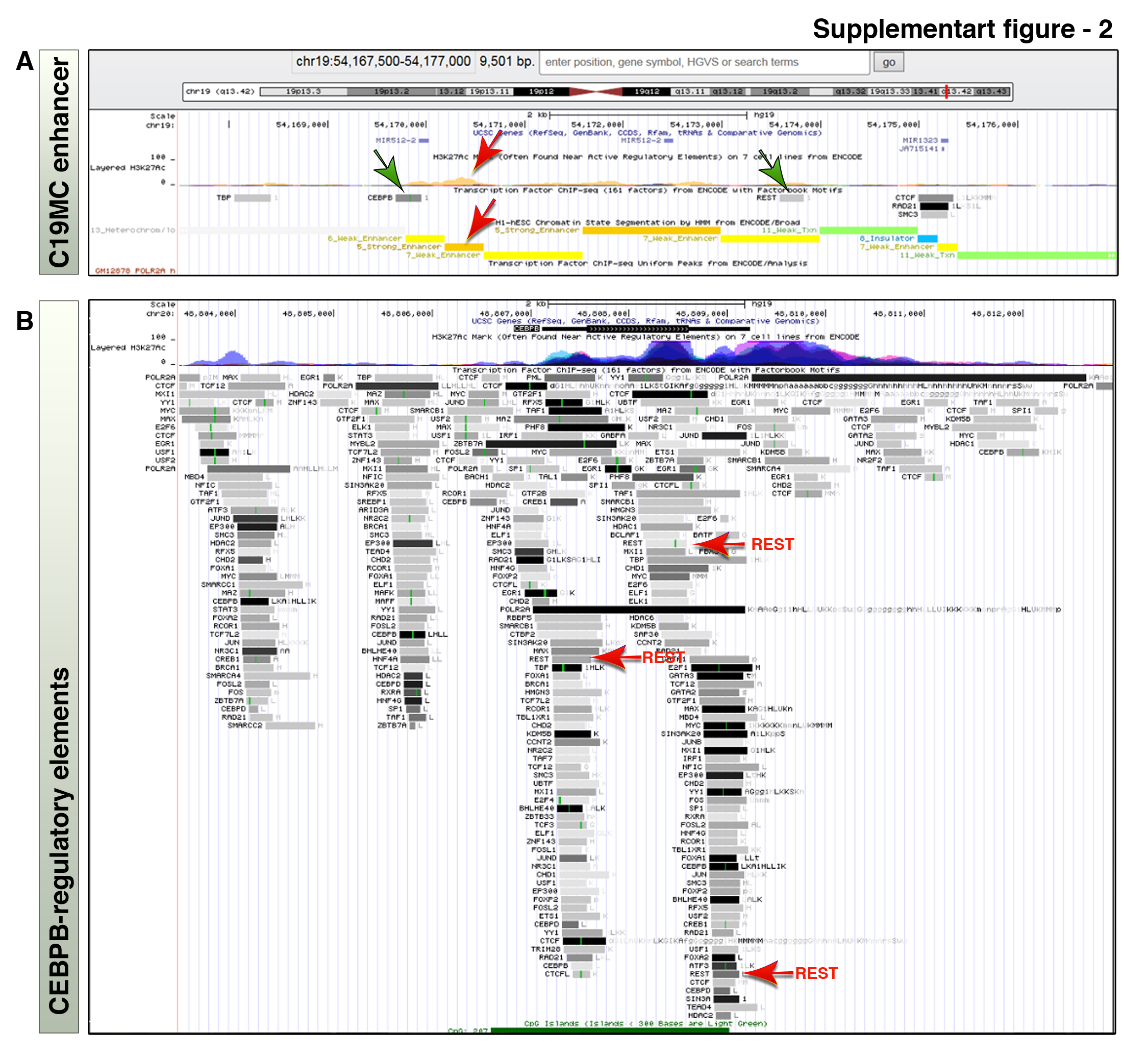

Supplement: S2 Fig — (A) UCSC genome browser (hg19) was used to examine the C19MC region. Green arrows show the CEBPB and REST binding sites and red arrows show the H3K27Ac mark and strong enhancer. (B) UCSC genome browser (hg19) was used to examine the CEBPB gene. Red arrows show the REST binding sites. Note the strong enhancer mark H3K27Ac (blue to purple/pink peaks). (TIF) [file pone.0206008.s002.tif]

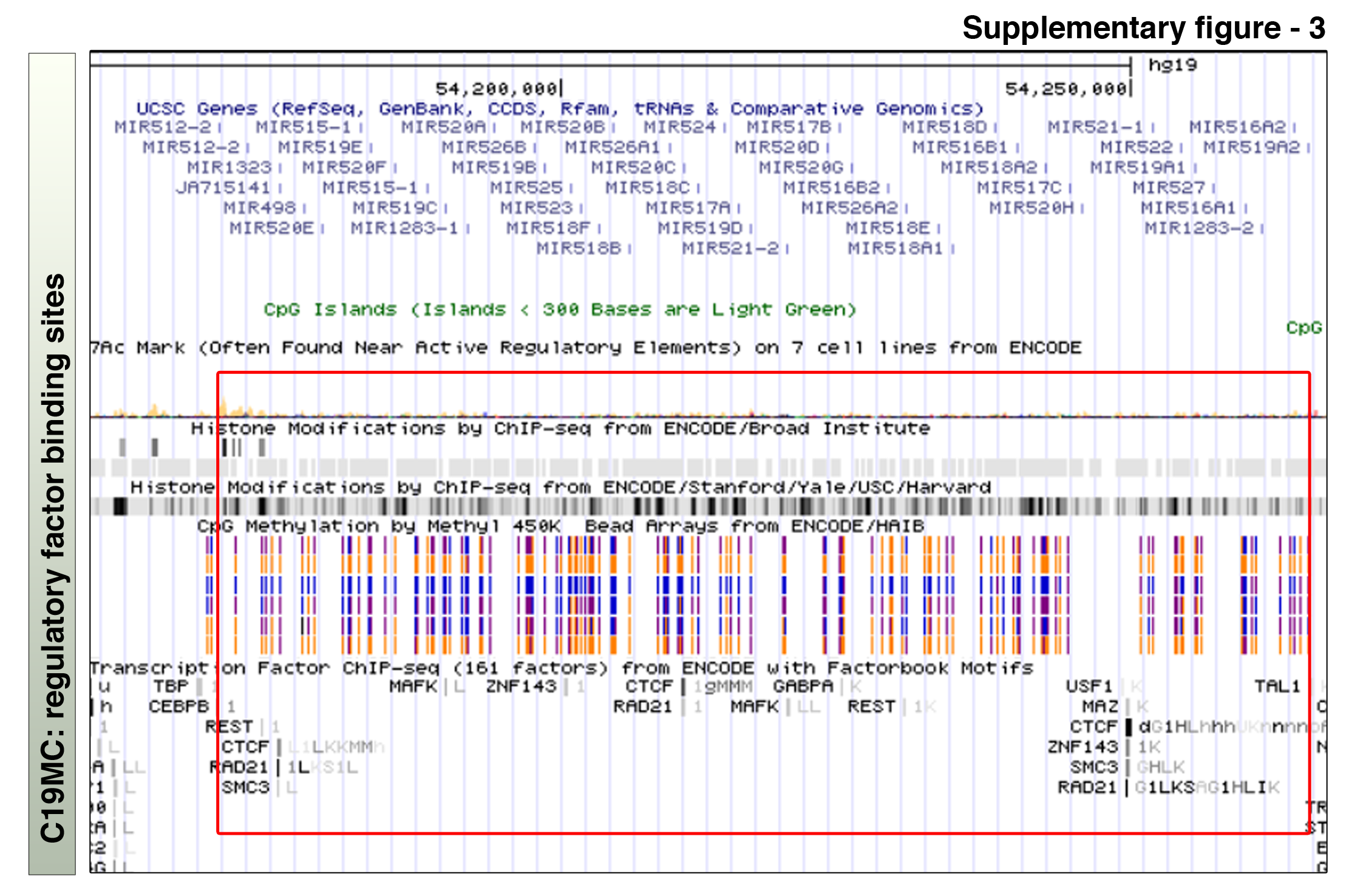

Supplement: S3 Fig — UCSC genome browser (hg19) was used to examine the transcription regulatory factor binding patterns in C19MC region (red box). Note the CEBPB and REST occupy the C19MC start site. (TIF) [file pone.0206008.s003.tif]

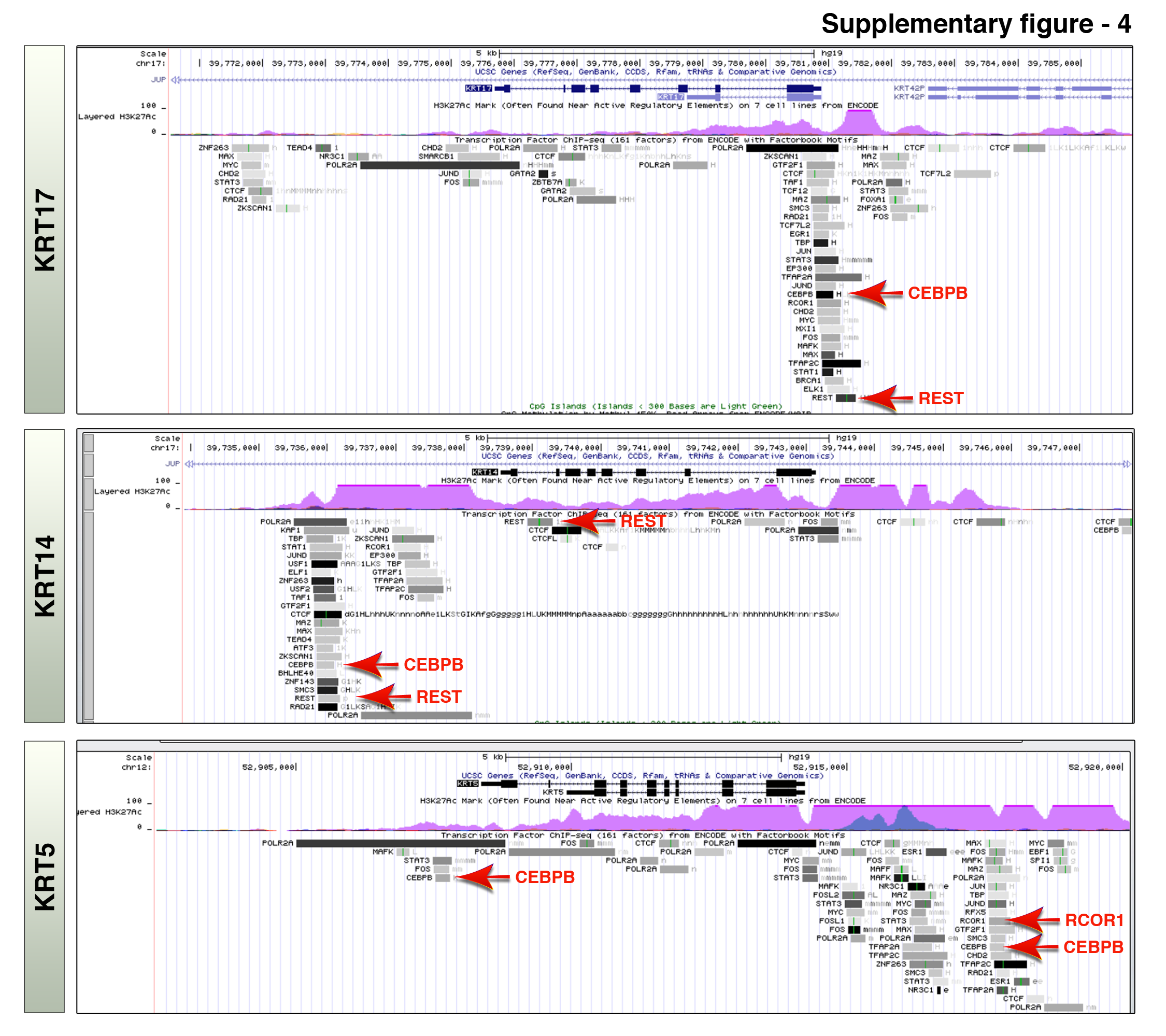

Supplement: S4 Fig — UCSC genome browser (hg19) was used to examine the transcription regulatory factor binding sites within within KRT17, KRT14 and KRT5 genes. Note the pink peaks (H3K27Ac marks) that indicating strong enhancer-mediated regulation of these genes. Red arrows indicate the REST binding sites or REST co-repressor RCoR1 binding sites. (TIF) [file pone.0206008.s004.tif]
