# Supplementary material for: Chromosome 19 miRNA cluster and CEBPB expression specifically mark and potentially drive triple negative breast cancers
Source: PLoS One. 2018 Oct 18;13(10):e0206008. doi: 10.1371/journal.pone.0206008 (PMC6193703; doi:10.1371/journal.pone.0206008)
Supplement: S1 File — (DOCX) [file pone.0206008.s005.docx]

**S1 File**

**Scripts for corrplots, color code and correlation efficiency**

> cor(); > mat <- cor(); > corrplot(mat, order = "hclust", addrect = 2, method = "color");

> col1 <- colorRampPalette(c("black", "white", "red"));

> corrplot(mat, order = "hclust", addrect = 2,, method = "color", col = col1(100))

Pearson’s correlation coefficiency plots were generated using script,

> col1 <- colorRampPalette(c("green", "black", "red")); > corrplot(mat, order = "hclust", addrect = 2, method = "number", col = col1(100)); where addrect = 2 was optional.
